# Comparison of an entirely automated ventilation mode, Intellivent-ASV, with conventional ventilation in ARDS patients: a 48-hour study

**DOI:** 10.1186/cc12036

**Published:** 2013-03-19

**Authors:** E Bialais, L Vignaux, X Wittebole, D Novotni, J Meyer, M Wysocki, T Sottiaux, G Reychler, J Roeseler, P Laterre, P Hantson

**Affiliations:** 1 Cliniques Universitaires Saint Luc, Brusells, Belgium; 2 Hamilton Medical AG, Bonaduz, Switzerland; 3 Clinique Notre-Dame de Grâce, Gosselies, Belgium

## Introduction

Intellivent-ASV has been developed to provide fully closed loop mechanical ventilation using a ventilation controller keeping EtCO_2 _and SpO_2 _within expert-based ranges. Ventilation of ARDS patients focuses on delivering adequate oxygenation and allowing elimination of CO_2 _while protecting the lung. The objectives were to compare Intellivent-ASV with conventional ventilation on safety and efficacy, and to compare the number of manual adjustments between the two ventilatory modalities.

## Methods

A randomized, controlled study including all consecutive patients receiving mechanical ventilation for at least 48 hours. Patients were randomly ventilated either with Intellivent-ASV or conventional ventilation, with a S1 (Hamilton, Bonaduz, Switzerland). Parameters were adjusted by the clinician in charge of the patient. Ventilatory and oxygenation parameters were recorded cycle by cycle during 48 hours and blood gases were performed every 6 hours.

## Results

Twenty-four patients with ARDS were included, 10 female, 14 male, median age 58 (46 to 63) years, APACHE II score 22 (17 to 29), PaO_2_/FiO_2 _at inclusion 136 (107 to 154). Eleven were ventilated in the conventional group and 13 in the Intellivent-ASV group. The study was stopped for one patient from the Intellivent-ASV group because of a pneumothorax not caused by ventilation. The delivered Vt was slightly higher during Intellivent-ASV (7.9 (7.5 to 8.5) vs. 7.2 (6.8 to 7.8) ml/kg, *P *= 0.013). The time spent by the various parameters in the suboptimal zone (safety) is the same for the two ventilation modes. The time spent in the optimal zone (efficacy) is the same for the two ventilation modes, expect for SpO_2 _(78% (75 to 89) with Intellivent-ASV vs. 44% (41 to 58), *P *= 0.001). Intellivent-ASV required less manual adjustments during 48 hours (2 (0 to 7) vs. 17 (10 to 27), *P <*0.001). The total number of adjustments during 48 hours, including automatic regulation, was higher in the Intellivent-ASV group (379 (274 to 493) vs. 17 (10 to 27), *P <*0.001).

## Conclusion

Over 48 hours, Intellivent-ASV allowed safe ventilation for ARDS patients, with better oxygenation and an identical ventilation efficacy when compared with conventional ventilation, with less manual adjustments.

